# Acute common iliac arterial occlusion caused by ruptured primary cardiac hydrated cyst -A case report

**DOI:** 10.1016/j.amsu.2019.08.003

**Published:** 2019-08-09

**Authors:** Samer Makki Mohamed Al-Hakkak, Ali N. Abed, Abbas k. Janabi, Muhanned K. Ali, Ahmed Abbodi Naema, Ahmed B. Mahdi

**Affiliations:** a Department of Surgery, Faculty of Medicine, Jabir Ibn Hayyan Medical University, Najaf City, Iraq; b Iraqi Center for Heart Diseases, Medical City Teaching Complex, Baghdad City, Iraq; c Department of Cardiovascular and Thoracic Surgery, Ghazi Al-Hariri Subspecialty Surgical Hospital, Medical City Teaching Complex, Baghdad, Iraq

**Keywords:** Embolism, Cerebral hydatid cyst, Rupture, Cardiac hydatid cyst, Echinococcosis

## Abstract

**Introduction:**

Hydatid cyst of the heart provides very rarely because of contractions of the heart natural resistance to the presence of viable cysts. Patients with cardiac hydatid cyst may remain asymptomatic for many years or have minor nonspecific complaints, but it is associated with an increased risk of lethal complications if left undiagnosed and untreated. Post rupture cardiac hydatid there is disseminated echinococcosis in the body.

**Case presentation:**

We report a healthy 20-year old male, while he was working as a farmer who presented with sudden onset acute right lower limb ischemia caused by ruptured of primary hydatid cyst of the heart which managed as a surgical emergency.

**Discussion:**

Patients with a cardiac hydatid cyst usually have symptoms after its rupture. Hydatid cyst rupture into left-sided chambers may cause systemic emboli, like in our case which presented with emboli of right common iliac artery and later on the patient had cerebral hydatid. Cardiac hydatid cyst can have two extremes, either remain asymptomatic over long periods or be discovered after serious and even fatal complications.

**Conclusion:**

Acute limb ischemia should be considered as an extremely a rare cause of rupture cardiac hydatid in an endemic area of echinococcosis also, this case confirms the need for the evaluation of the patient well after ruptured cardiac hydatid for other sites of emboli like brain hydrated as in our patient.

## Highlights

• Cardiac hydatid cyst is rare; rupture cardiac hydatid is lethal.
• Complication should be considered in case of ruptured cardiac hydatid, even later on.
• Rupture cardiac hydatid lead to disseminated emboli that lodged in any part of the body, leads to serious complications.

## 1 Introduction

Hydatid cyst (HC) disease is a serious health problem in endemic areas, Humans become infested with the parasite by ingesting embryonated eggs shed in dogs and other canids’ faeces [1,2]. Embryos are released in the small intestine and reach different organs through the bloodstream. The larval stages emerging from the eggs develop into hydatid cysts in the liver (65%), the lungs (25%), the muscles (5%), or the heart (0.5%–2%) [3]. Although infestation of the heart with the parasite is rare if occurs, it can lead to severe complications and is often fatal when left untreated. Embryos reach the right atrium of the heart through the thoracic duct or the inferior vena cava and then pass to the right ventricle and onto the pulmonary artery [4]. They can also reach the myocardium through the pulmonary vessels and coronary arteries. Cysts can be found in any part of the heart, and they are known to grow in slow, intermittent stages over many years [5,6]. Although at first asymptomatic, hydatid cysts of the heart can cause symptoms at later stages, which will vary depending upon the location of the infestation within the heart. Published articles and case reports related to cardiac hydatid cyst (CHC) are limited, and many of questions remain unanswered. In most recorded cases of CHC, a correct diagnosis of the condition was made only at postmortem examination. Arterial involvement with echinococcosis is quite rare [7,8]. Rupture into the cardiac chambers may result in systemic or pulmonary embolization. Our case we describe two complications of rupture CHC peripheral artery embolization, ischemic limb and later on cerebral hydatid. The work has been reported in line with the SCARE criteria [9].

## 2 Case presentation

**Fig. 1:**
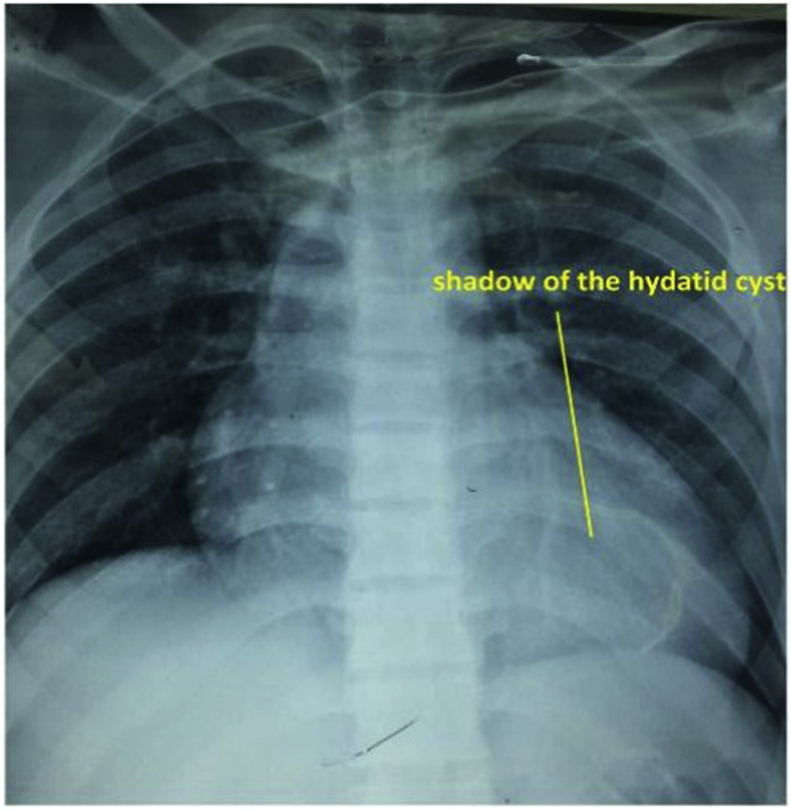
Preoperative CXR shadow of Hydatid cyst.

**Fig. 2:**
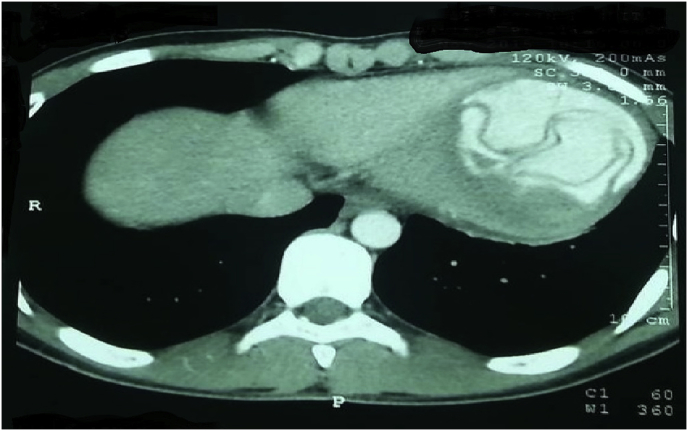
CT Scan show laminated membrane ruptured LV Hydatid cyst.

**Fig. 3:**
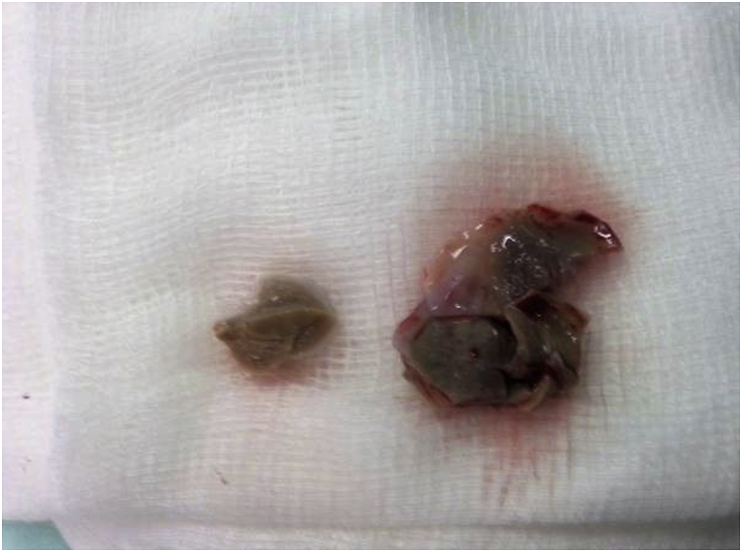
Hydatid cyst fragments from retrieved common iliac artery.

**Fig. 4:**
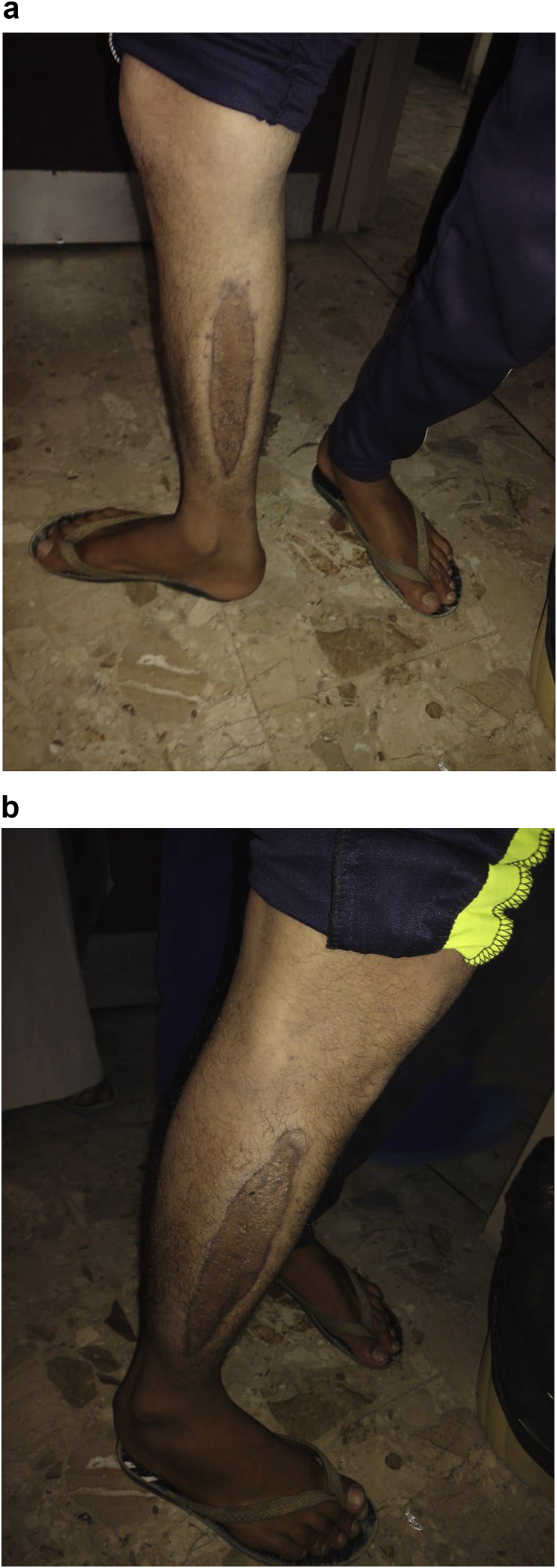
a Fasciotomy of the medial aspect of the Rt lower leg. 4b.Scar of lateral aspect of right leg fasciotomy post embolecty.

**Fig. 5:**
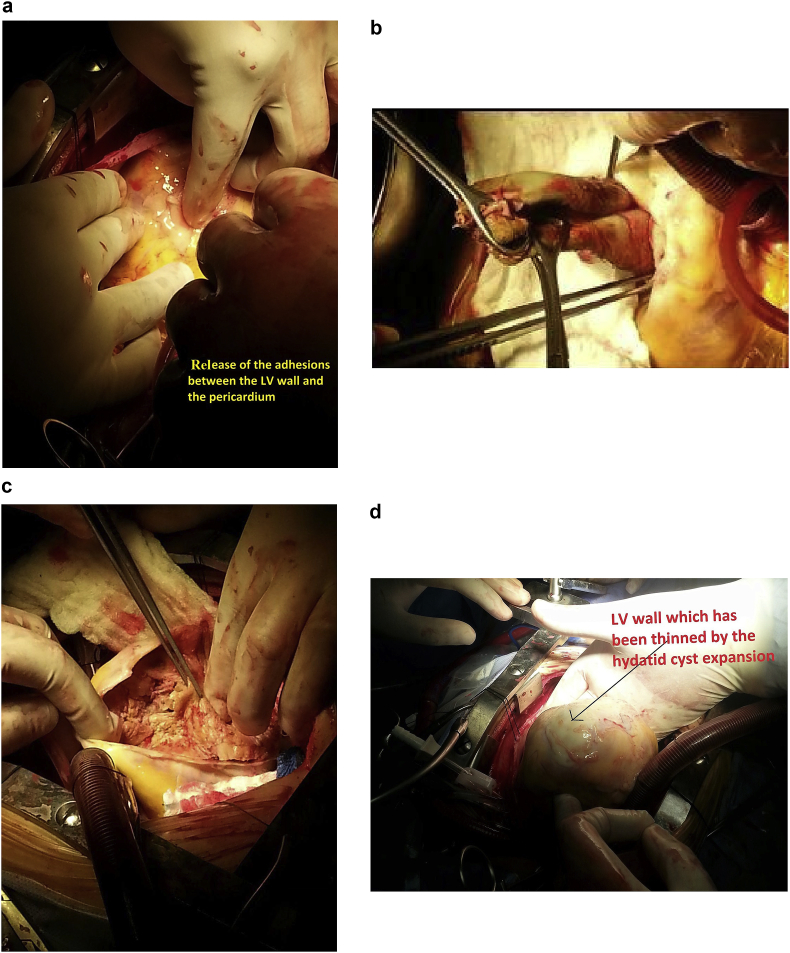
a The release of adhesions between the LV wall and pericardium. 5b.Hydatid cystic wall extraction from Heart. 5c.Debridement of the cavity_spl_ commuication between LV and cyst. 5d.LV wall which has been thinned by the hydatid cyst expansion.

**Fig. 6:**
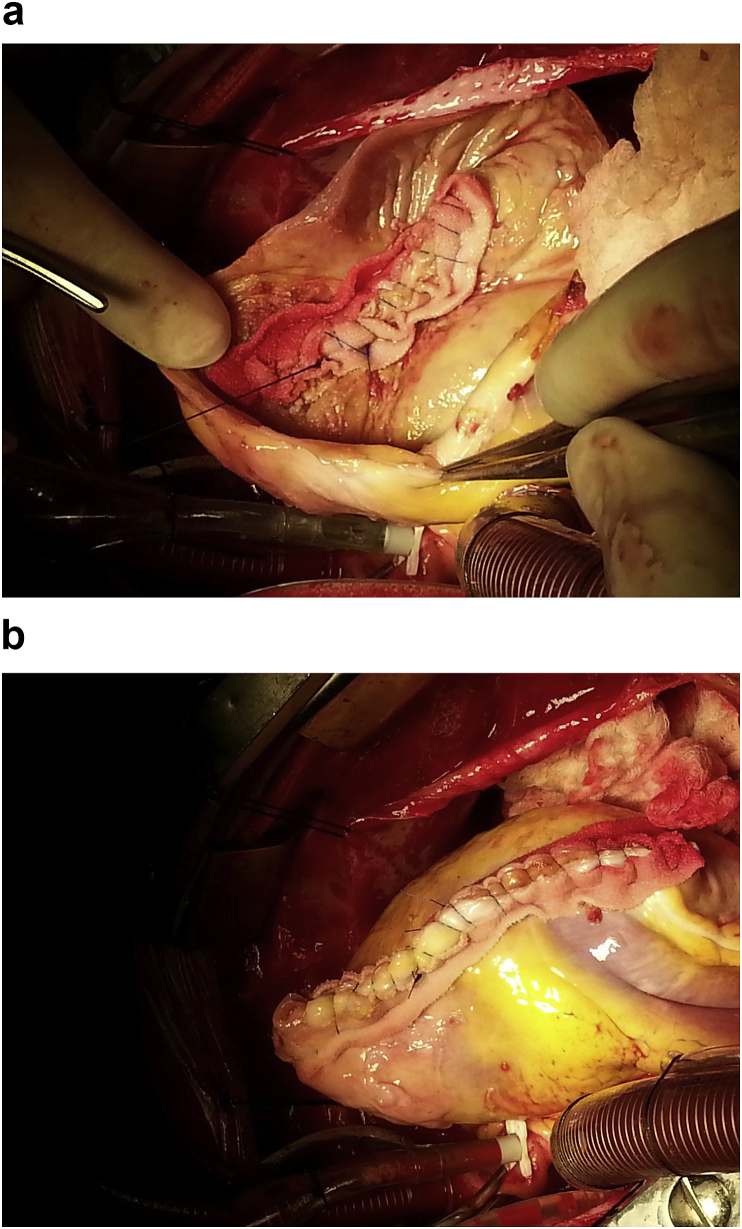
a First layer closure of the LV. 6b.Second layers closure of LV.

**Fig. 7:**
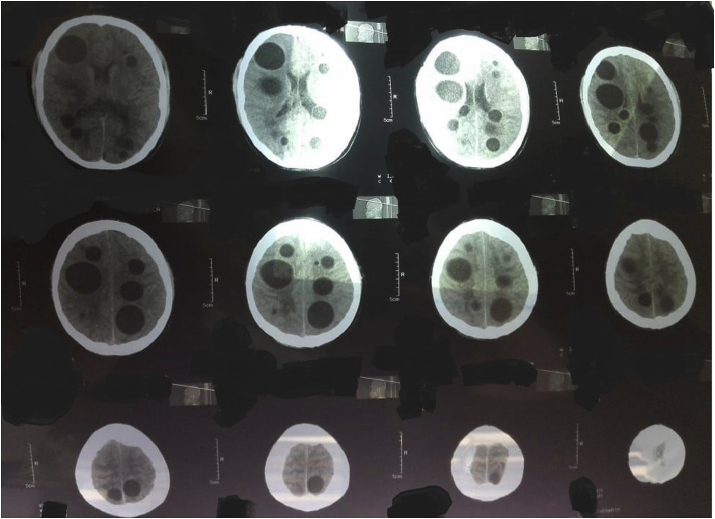
CT Scan brain 11 months postoperative open heart surgery.

**Fig. 8:**
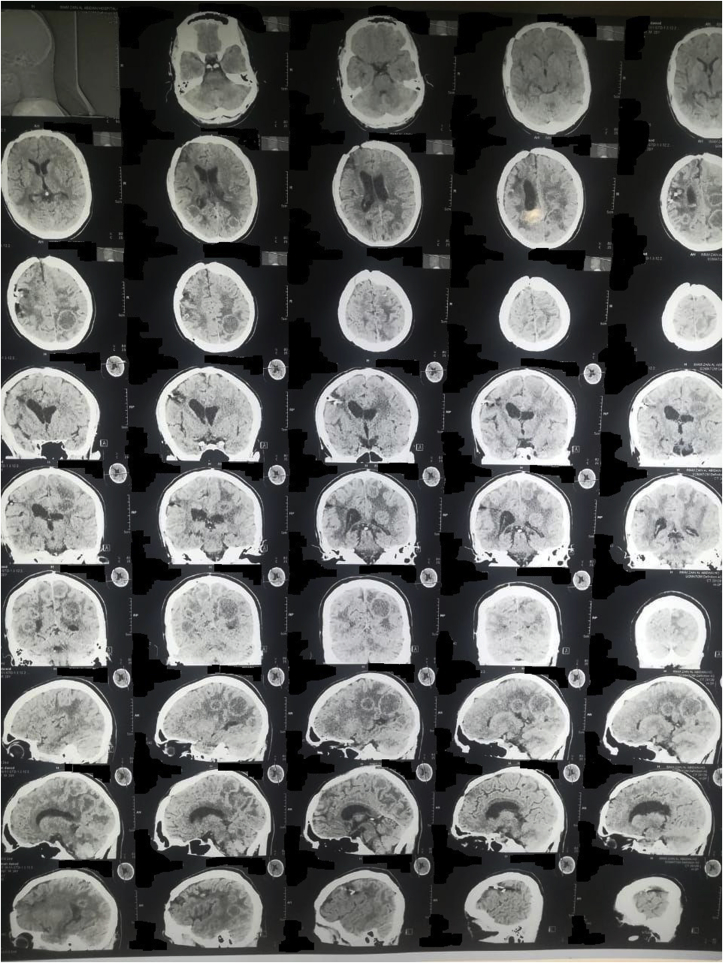
CT Scan brain 10 months postoperative brain surgery.

A 20-year-old man farmer whose family lived in a rural area healthy, nonsmokers no history of any drug taken without any congenital anomalies were admitted to our hospital with sudden-onset pain, absent pulses, paresthesia, and the pallor of the right lower extremity that had begun two hours before admission while he was working on the farm. The pain had been increasing and a change in color of the affected right lower limb also was associated with severe headache tachycardia and irritability. An urgent Doppler ultrasound is done for lower abdomen and right lower limb which show the right common iliac artery with hypoechoic acute thrombus inside with near-complete obliteration of flow, then the patient sent for echocardiogram study show left ventricular collapse with pericardial cystic mass with thrombus inside the cyst. Chest X-ray did show the shadow of cardiac hydatid cyst as shown in Fig. 1 then suspicious of rupture cardiac hydatid confirmed by sending the patient for computerized (CT) scan of the chest with show complicated solitary cystic mass of the LV mostly hydatid cyst as shown in Fig. 2 The patient was referred to a tertiary center to complete his management, the patient presented in a state of hemodynamic instability, central nervous system irritability, chest pain, anuria, and elevated renal indices. The patient needed urgent surgical intervention, we take the consent and inform the relative of the patient about risk and urgency. Firstly embolectomy was done as an emergency, embolus (Fragments of the rupture infected cyst) retrieved from the right common iliac artery as shown in Fig. 3 then open fasciotomy post embolectomy done because of a sign of compartment in legs happen as shown in Fig. 4a and b then the patient sent for open-heart surgery for removal of the ruptured cardiac hydatid cyst which was occupying the left ventricular apex (about the size of a tennis ball) and before of opening wall of LV injection of hypertonic saline 30%, which safe, to minimize or prevent hydatid fluid and scolices to systemic dissemination, after cystic wall opening, removal of cyst wall found connecting the true Left ventricle (LV) cavity to the ruptured cyst as shown in Fig. 5a, b, c, and d. Double layer closure of the left ventricular wall as shown in Fig. 6a and b. Smooth weaning from bypass patient sedated for 3 days in the intensive care unit, full neurologic and renal recovery, and fairly preserved LV function 7 days postoperatively. CT scanned of brain, chest, and abdomen was performed for possible dissemination of other parts of the body. There was no evidence of any another cyst so its primary LV hydatid cyst. Keep the patient on albendazole 400mg twice a day for about 6 months, continues to follow up, 11 months later the patient presented with agitation and drowsiness, so a brain CT scan did show multiples small and large brain hydrated as shown in Fig. 7 Patient start another course of albendazole 400mg twice a day for one week which then surgery is done for larger cysts, while small cysts continue on the same regime of albendazole 400mg twice daily for 3 months every 28 days albendazole regime we wait 7 days then start another regime, patient continue albendazole treatments and follow up of the small hydatid cyst as shown in CT scan of bran 10 months post cerebral hydatid surgery Fig. 8 The condition of the patient well, continue monitoring, most of the small cyst regress but still keep the patient on regular monitoring for watch further complication and deals accordingly.

## 3 Discussion

Hydatid cysts of the heart are usually primary, and these primary cysts are solitary as in our case. If multiple cysts are found, then they are secondary after rupture. Larva reaches the right side of the heart through the thoracic duct and superior vena cava; from the right ventricle, the embryo passes through the pulmonary capillaries into the left ventricle, from where it could reach any part of the body through the systemic circulation. Some authors have suggested transmigration of the embryo through the interatrial and interventricular septum to the left side of the heart. Larvae reach the myocardium through the coronary circulation. A hydatid cyst involving the heart has the following predominant locations: left ventricle (75%) this can be explained by this cardiac site's high vascularization and muscle bulk, right ventricle (18% less muscle bulk and vascularization), and interventricular septum (7%) [10]. With cysts on the left side of the heart death may occur from rupture of the ventricle or intracardiac rupture of the cyst leading to cerebral embolism or anaphylactic shock. Hydatid cysts were mostly located in the ventricular wall, rather than in the lumen because high intraventricular blood pressure reduces the chance of seeding of the hydatid cysts in the latter [11,12] Left ventricular hydatid cyst are usually located subpericardially and rarely rupture into the pericardial space.

The clinical picture of CHC depends on the localization, age, size, and quantity of the cysts and the degree of calcification. Persons with a cardiac hydatid cyst may be asymptomatic, though such a presentation is rare. Patients with a cardiac hydatid cyst usually have symptoms after its rupture. Sudden death caused by anaphylactic shock may occur after the rupture of a cyst [13]. The rupture may be silent, and metastatic echinococcosis of various organs may be a late—and often sole—clue to the presence of an underlying cardiac involvement [14]. Rupture CHC lead to embolization of cysts to the large vessels was a rare complication of this infectious disease. Depending on the site of origin, emboli can lodge anywhere in the vascular tree. In this case, hydatid cysts originated from the LV of the heart and moved through the abdominal aorta to all over the vessels of the body, which is compatible with the definition of emboli. The diagnosis of hydatid cyst of the heart is difficult because of clinical and also radiographic findings may be nonspecific [15]. The embryo usually reaches the myocardium via the coronary circulation from the left side of the heart or reaches the epicardium via lymphatic circulation [6,16].

Cysts can be found in any part of the heart, and they are known to grow at slow, intermittent stages over many years. The therapy of hydatidosis consists of surgical removal of the cyst and administration of albendazole (10–15 mg/kg per day) for 3–6 months, with a maximum dose of 800 mg [17]. The rupture of a left-sided hydatid cyst may result in systemic emboli like out case-patient had cerebral hydatid later on. These cysts are usually multiple, in contrast to primary cysts of the central nervous system that are mostly solitary. So secondary multiple hydatid cysts of the central nervous system caused by cardiac embolization are infertile [18]. our patient gets secondary cerebral hydatid from rupture CHC, there is no consensus on the growth rate of the hydatid cysts of the brain and has been variably reported between 1.5 and 10 cm/year [19].

The marked incidence of catastrophic complications of cardiac hydatid cyst emphasizes the need for early diagnosis. Investigation of the hydatid cysts of the heart should always be carried out and considered carefully in a heavily infested area of the world such as our country.

## 4 Conclusion

Cardiac hydatid cyst is rare and serious. Late diagnosis of a CHC is responsible for poor prognosis linked to the rupture risk, the emboli dissemination which is an emergency status, embolectomy, and open-heart surgery is necessary. Although acute arterial embolism is a rare form of atypical presentation of disseminated echinococcosis, in our country, an endemic area for echinococcosis, it should be considered as a differential diagnosis for arterial thromboembolism or cardiac mass in patients with compatible imaging and epidemiology. Surgery-associated with medical treatment provides good results but later on complication like brain hydatid as demonstrated in our case report. So continues monitoring of the patient is mandatory.

## Ethical approval

The study is exempt from ethical approval in my institution.

## Funding

Non funding.

## Author contribution

**Samer Makki Mohamed Al-Hakkak**: Conception and design of study, Writing paper and data collection, Informed Consent, Data supplements and pictures, Drafting of manuscript and or revision /final revision for approval; **Ali N. abed**: Data supplements and pictures, Leader Team Cardiothoracic surgeon who do open heart surgery; **Abbas k. janabi**: Data supplements and pictures, Leader team cardiothoracic surgeon who do embolectomy; **Muhanned K. Ali**: Cardio thoracic surgeon, assisted in embolectomy; **Ahmed Abbodi Naema**: Cardiovascular and thoracic surgeon, assisted in open heart surgery; **Ahmed B. Mahdi**: Specialist Cardiac anesthetist, gave anesthesia for

## Conflicts of interest

No any conflicts of interest.

## Research registration number

Researchregistery4740.

## Guarantor

Samer Makki Mohamed Al Hakkak

## Consent

Written informed consent was obtained from the patient for the publication of this case report and the accompanying images.

## Provenance and peer review

Not commissioned, externally peer reviewed.
